# Factors affecting pre- and post-stenting computed tomography perfusion in patients with middle cerebral artery stenosis

**DOI:** 10.3892/etm.2012.805

**Published:** 2012-11-09

**Authors:** HUI QU, JINLI LI, XINGQUAN ZHAO, KEHUI DONG

**Affiliations:** Department of Neurology, Beijing Tiantan Hospital affiliated to Capital Medical University, Beijing 100050, P.R. China

**Keywords:** computed tomography perfusion, time to peak, stent, Alberta stroke program early computed tomography scoring

## Abstract

The aim of this study was to investigate the factors affecting pre- and post-stenting head computed tomography perfusion (CTP) in patients with middle cerebral artery stenosis. A total of 25 patients with severe middle cerebral artery stenosis were enrolled. CTP was performed prior to and following stenting. Scores were allocated to the time-to-peak (TTP) parameter of CTP using the Alberta stroke program early computed tomography scoring (ASPECTS) scale. The factors possibly affecting pre- and post-stenting CTP were analyzed. All the patients exhibited markedly prolonged TTP on the affected side prior to stenting, compared with the healthy side. Following surgery, the TTP was improved in all patients. The preoperative ASPECTS score was negatively correlated with the degree of middle cerebral artery stenosis with a correlation coefficient of −5.78. The preoperative vascular stenosis rate was positively correlated with the improvement degree of the ASPECTS score with a correlation coefficient of 1.137 (P=0.001). TTP is a sensitive parameter for evaluating the effect of stenting on middle cerebral artery stenosis. TTP prior to and following stenting may be quantitatively assessed using the ASPECTS scale. Patients with serious stenosis and/or good collateral circulation are able to benefit more from stenting.

## Introduction

The head computed tomography perfusion (CTP) technique is an important method for cerebral blood flow assessment (1–4). This technique visually represents the blood flow perfusion in various parts of the brain tissue. In the application of CTP, time to peak (TTP) is the most sensitive and specific index for brain ischemia (5). Stenting is a technique which has been applied in cerebrovascular disease treatment, particularly for ischemic cerebrovascular diseases (6–10). The effect of stenting on ischemic cerebrovascular disease may be evaluated visually using the CTP technique (11,12).

In the present study, the Alberta stroke program early computed tomography scoring (ASPECTS) scale was used to quantitatively analyze CTP in 25 patients with ischemic cerebrovascular disease caused by middle cerebral artery solitary stenosis prior to and following stenting. The factors affecting CTP prior to and following stenting were further analyzed (13,14).

## Patients and methods

### Clinical data

A total of 25 patients with cerebrovascular disease caused by unilateral middle cerebral artery solitary stenosis that received treatment at Tiantan Hospital (Beijing, China) between January 1, 2004 and March 1, 2007 were enrolled. All patients were subjected to percutaneous stent implantation (stenting). Head CTP imaging was performed within 7 days before and after stenting. Of the 25 patients, 12 had transient ischemic attack (TIA) and 13 had cerebral infarction (CI). The patient’s ages ranged between 34 and 71 years with an average of 49.4±8.4 years. Of the patients, 19 were male and 6 were female. The degree of stenosis ranged between 60 and 99% with an average of 80.1±11.4%. This study was conducted with approval from the Ethics Committee of Capital Medical University. Written informed consent was obtained from all participants.

### Pre-stenting evaluation

Preoperative evaluation included angiographic classification of the cerebral arterial stenosis based on Mori’s method (15,16), clinical typing of the cerebral arterial stenosis (6) and classification based on whether apparent cerebral blood flow collateral circulation existed (17).

### CTP examination

CT scanning was performed using a GE Lightspeed spiral CT system. The scanning matrix was 512x512 and the exposure conditions were 120 kV and 100 mA. Successive scanning of the layers of interest began when a high pressure injector was started for rapid intravenous injection of the contrast medium. The slice thickness was 10 mm and the scanning lasted 60 sec at a 1 slice/sec scanning rate (60 slices in total). Iohexol (300 mg/ml) was used as the contrast medium, with a volume of 40 ml at an 8 ml/sec flow rate. The layers of interest included the basal and coronal radiation layers. The regional cerebral blood flow (rCBF), regional cerebral blood volume (rCBV), mean transit time (MTT) and TTP parameters were calculated using professional software. Color maps were obtained.

### Pre- and post-stenting CTP scoring criteria

The CT parameter color maps prior to and following stenting were compared and the head CTP TTP prior to and following stenting was scored using the ASPECTS scale (13,14). The highest possible mark of the scale is 10 points. Higher scores indicates greater cerebral perfusion. The TTP improvement degree was then calculated based on the following formula: TTP improvement degree = (post-stenting ASPECTS score - pre-stenting ASPECTS score)/10x100(%).

### Statistical analysis

The χ^2^ test was used to compare categorical data, and t-tests or paired t-tests were used to compare continuous data. Correlation analysis and linear regression were performed to calculate the correlation coefficient and regression equation. All data were analyzed using SPSS 11.5 statistical software.

## Results

**Figure 1.**
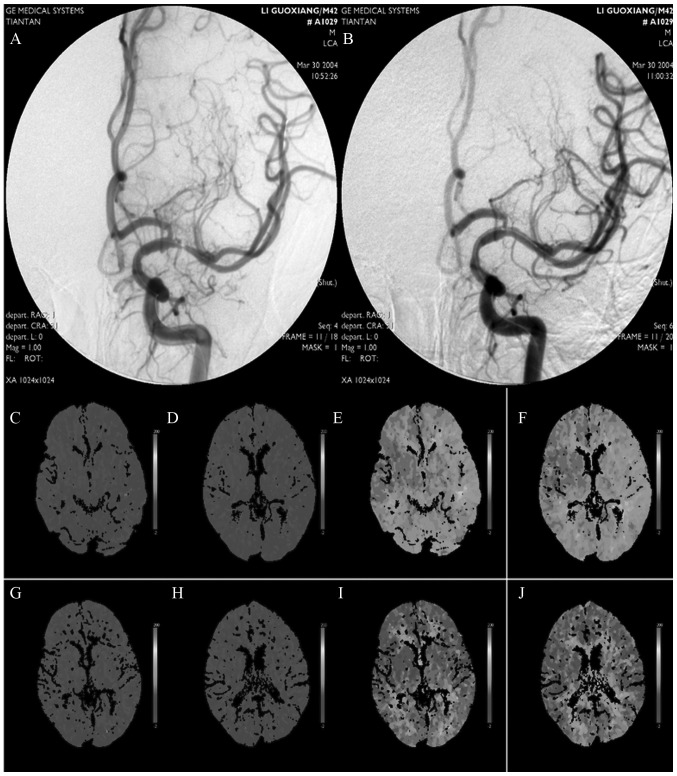
DSA and CT images of patients with middle cerebral artery stenosis. (A) Preoperative stenosis rate of 99% at the M1 segment of the LMCA by DSA; (B) no notable postoperative residual LMCA stenosis in the same patient by DSA; (C–F) preoperative prolonged MTT and TTP at the left frontal lobe, left temporal and bilateral occipital lobes, respectively, by cranial perfusion CT; and (G–J) postoperative, essentially normal MTT and TTP at the left frontal and temporal lobes but high MTT and TTP at the bilateral occipital lobes. LMCA, left middle cerebral artery; DSA, digital subtraction angiography; MTT, mean transit time; TTP, time-to-peak.

The CTP color maps revealed that the TTP values in all the 25 patients were significantly prolonged prior to surgery, while following surgery, all these values improved by various degrees. The ASPECTS score (mean ± SD) prior to surgery was 2.32±1.31, whereas that following surgery was 8.28±1.65, indicating a significant difference (P<0.01). The preoperative ASPECTS score was negatively correlated with the degree of middle cerebral artery solitary stenosis with a correlation coefficient of −5.78 (Fig. 1).

Furthermore, the correlation factors possibly affecting pre- and post-stenting CTP improvement were analyzed and compared between the subgroups. These factors included gender, discharge diagnosis of manifestations and vessels (including CI and TIA), collateral circulation, histories of high blood pressure, diabetes, coronary heart disease and smoking, as well as dyslipidemia and hyperhomocysteinemia. The comparisons revealed that only the collateral circulation subgroups exhibited a significant difference (P=0.033). The results are shown in Table I. Correlations of age, the preoperative vascular stenosis rate, postoperative residual stenosis rate and pre- and post-operative scores based on the National Institutes of Health stroke scale (NIHHS) with CTP improvement were analyzed. The results revealed that only the preoperative vascular stenosis rate was positively correlated with the CTP ASPECTS score improvement with a correlation coefficient of 1.137 (P=0.01; Table II).

## Discussion

Cerebral arterial stenosis is a significant pathological mechanism leading to ischemic cerebrovascular disease. Theoretically, the removal of stenosis and improvement of the cerebral blood flow is likely to decrease the incidence of CI. Percutaneous endovascular stenting has been demonstrated to be an effective treatment method for intracranial cerebral arterial stenosis (6–8), although evaluations of the effectiveness of stenting are often based on long-term stroke and preoperative event incidence rates (6,7). CTP is highly sensitive to the improvement effect of stenting on cerebral perfusion. CTP color maps visually represent the cerebral perfusion improvement following stenting (12,18) but are not contributory to scientific research statistics. The ASPECTS scale was an early tool for evaluating the effect of thrombolytic therapy on CI (13,14). Since the radiological layers involved in the ASPECTS scale are the same as the CT scanning layers involved in the CTP technique, the scale may also be used for evaluating middle cerebral arterial blood supply.

All 25 patients in the present study had middle cerebral artery solitary stenosis. The CTP color maps revealed that their TTP was prolonged prior to stenting, whereas following stenting, the values were improved significantly. These findings were consistent with those reported previously (11,12). The ASPECTS score also revealed a significant difference in the cerebral perfusion prior to and following stenting (P<0.01), which indicated a perfusion improvement following stenting. This result suggests that the evaluation of CTP improvement using the ASPECTS scale is feasible. However, although the ASPECTS score bore a negative correlation with the degree of severity of middle cerebral stenosis prior to stenting, a correlation was not observed between the ASPECT score and residual stenosis following stenting. When the middle cerebral stenosis was between 60% and 99%, the more serious stenosis led to a lower ASPECT score, whereas when residual stenosis fell below 30%, the ASPECT score did not appear to vary according to the severity of stenosis. This suggests that the ASPECTS scale has statistical significance only within certain degrees of stenosis.

Furthermore, the correlation factors which may affect CTP prior to and following stenting were also analyzed in the present study. The studied factors included age, gender, histories of high blood pressure, smoking, drinking, diabetes and coronary heart disease, as well as hyperlipidemia, hyperhomocysteinemia, postoperative residual stenosis rate, NIHHS score prior to and following surgery and discharge diagnosis (TIA and CI). The analysis revealed that the preoperative vascular stenosis rate and collateral circulation were the only factors correlated with the degree of CTP improvement. This result suggests that the non-correlated factors are negligible in surgical patient selection and prognosis assessment. The preoperative vascular stenosis rate was markedly correlated with the degree of postoperative CTP improvement. A more serious degree of stenosis indicated a more marked improvement effect of stenting for cerebral perfusion and cerebral ischemia. In addition, whether there was good collateral circulation in the blood supply region prior to stenting also greatly affected the degree of postoperative CTP improvement. Patients without good collateral circulation exhibited greater degrees of CTP improvement. This suggests that patients without good collateral circulation benefit more from stenting, which is in agreement with the reported literature (7).

Based on the findings of the present study, the degree of vascular stenosis and whether good collateral circulation exists should be considered first when stenting is selected for patients with intracranial arteriostenosis, in order to achieve satisfactory cerebral perfusion improvement. The considerations of age, gender, drinking, high blood pressure, diabetes, coronary heart disease, lipid disorders and hyperhomocysteinemia should then be considered. However, the present study had a significant limitation. In order to remove the effects of external factors, all the recruited patients were those with single cerebral artery disease. The patients received CTP detection prior to and following stenting. Due to the strict selection criteria, the sample size in the present study was small with only 25 subjects enrolled.

## Figures and Tables

**Table I.** T-test analysis of the factors affecting pre- and post-operative CTP.

Factor	Pre-stenting ASPECTS	Post-stenting ASPECTS	Improvement degree (%)	P-value
Gender				0.121
Male	2.26±1.33	8.58±1.50	63.16±17.34	
Female	2.50±1.38	7.33±1.86	48.33±26.40	
High blood pressure				0.782
Yes	2.36±1.03	8.45±1.75	60.91±20.72	
No	2.29±1.54	8.14±1.61	58.57±20.70	
Diabetes				0.462
Yes	1.50±0.71	8.50±2.12	70.00±14.14	
No	2.39±1.34	8.26±1.66	58.70±20.74	
Coronary heart disease^a^				0.315
Yes	2.00	10.00	80.00	
No	2.33±1.34	8.21±1.64	58.75±20.28	
Smoking				0.426
Yes	2.17±1.10	8.33±1.50	61.67±18.23	
No	2.71±1.80	8.14±2.12	54.29±25.73	
Drinking				0.384
Yes	2.50±1.17	8.08±1.51	55.83±18.32	
No	2.15±1.46	8.46±1.81	63.08±22.13	
Dyslipidemia				0.190
Yes	2.00±0.93	8.40±1.80	64.00±19.57	
No	2.80±1.69	8.10±1.45	53.00±20.58	
Hyperhomocysteinemia				0.484
Yes	2.43±1.27	8.86±1.21	64.29±19.02	
No	2.28±1.36	8.06±1.76	57.78±21.02	
Discharge diagnosis				0.286
TIA	2.83±1.47	8.33±1.61	55.00±21.95	
CI	1.84±0.99	8.23±1.74	63.85±18.50	
Collateral circulation				
Yes	2.91±1.38	7.91±2.02	50.00±21.91	0.033
No	1.86±1.10	8.57±1.28	67.14±15.90	

CTP, computed tomography perfusion; ASPECTS, Alberta stroke program early computed tomography scoring; TIA, transient ischemic attack; CI, cerebral infarction.

a One patient with coronary heart disease was analyzed.

**Table II.** Correlation analysis of the factors possibly affecting pre- and post-stenting CTP.

Factor	P-value
Age	0.839
Preoperative vascular stenosis rate	0.001
Postoperative residual stenosis rate	0.923
Pre-stenting NIHHS score	0.668
Post-stenting NIHHS score	0.596

CTP, computed tomography perfusion; NIHHS, National Institutes of Health stroke scale.
